# Supplementary material for: Critical Competences for the Management of Post-Operative Course in Patients with Digestive Tract Cancer: The Contribution of MADIT Methodology for a Nine-Month Longitudinal Study
Source: Behav Sci (Basel). 2022 Apr 9;12(4):101. doi: 10.3390/bs12040101 (PMC9029589; doi:10.3390/bs12040101)
Supplement: Supplementary file 1 [file behavsci-12-00101-s001.zip › Table S1_def.pdf]

Table S1. HEAGIS-DQ questions for each competence and area.

Area	Competence	Question
Clinical Area	Preview of future scenarios	1) We ask you to imagine that you are at the control visit after about 3 months from the surgery: the surgeon will ask you to describe your physical-health condition after the surgery: what could be your answers?
	Use of resources	2) Consider the answer you gave to the previous question: after discharge from the hospital for the surgery you underwent, what roles could help you and how?
	Situation evaluation	3) Once discharged from the hospital, what can be the way to manage the following period?
	Preview repercussion of the own actions	4) We ask you to imagine that you are 3 months from now in a situation where you have to choose whether or not to do a certain thing. However, you are not sure whether this is in line with the instructions given to you by the medical staff. On what basis would you make the decision?
Everyday Activities Area	Preview of future scenarios	5) We are now asking you instead to think about your hobbies, your passions, the daily activities you do in life. How will you be able to do them 3 months after surgery?
	Situation evaluation	6) Regarding your answer to the previous question, what did you consider in answering it?
	Preview repercussion of the own actions	7) Think about 3 activities, among those you do in your daily life, that you will pay particular attention to because of the effects of the surgery. Why did you think about these 3 activities and not others?
	Use of resources	8) How would you cope with any difficulties in your daily activities after discharge?
Family Area	Preview of future scenarios	9) Imagine that you have just come home from your inpatient stay and that 3 months have passed since now. How would you describe these 3 months?
	Situation evaluation	10) We ask you to project yourself 3 months after discharge from the hospital: a friend asks you how things are going with your family. What would you take into consideration in responding to the friend?
	Preview repercussion of the own actions	11) Thinking about projecting again at 3 months after discharge, how will you be contributing to the family's life?
	Use of resources	12) What did you consider in answering the previous question?
Job Area	Preview of future scenarios	13) Now consider your current job: what will it be like to start over after surgery?
	Situation evaluation	14) And how will you manage the return to your work activity?
	Preview repercussion of the own actions	15) Consider a typical working day: do you think it will need to be changed because of the surgery you have undergone? On what basis did you answer?
	Use of resources	16) Still regarding a typical day at work, please think about a situation in which you might find yourself in trouble: how would you handle it?
