# Supplementary material for: Critical Competences for the Management of Post-Operative Course in Patients with Digestive Tract Cancer: The Contribution of MADIT Methodology for a Nine-Month Longitudinal Study
Source: Behav Sci (Basel). 2022 Apr 9;12(4):101. doi: 10.3390/bs12040101 (PMC9029589; doi:10.3390/bs12040101)
Supplement: Supplementary file 1 [file behavsci-12-00101-s001.zip › Table S2_def.pdf]

Table S2. Criteria for the definition of each level of competence.

Competence	Level of competence	Criteria for high/medium/low competence
Preview of future scenarios	HIGH	The patient can depict multiple scenarios which can occur, using shared criteria.
	MEDIUM	The patient can depict multiple scenarios which can occur, but uses only personal criteria and opinions.
	LOW	The patient depicts just one possible scenario, using personal criteria and opinions
Situation evaluation	HIGH	Context elements about present condition are used to manage evaluations or to describe the situation
	MEDIUM	Context elements are only mentioned, but not used to performs any sort of evaluation
	LOW	Context elements are either not mentioned or addressed using opinions and personal beliefs
Preview repercussion of the own actions	HIGH	The patient can depict multiple scenarios which can occur, using shared criteria, about implications of his/her actions
	MEDIUM	The patient can depict multiple scenarios which can occur, but uses only personal criteria and opinions to consider implications of his/her actions
	LOW	The patient depicts just one possible scenario, using personal criteria and opinions, about implications of his/her actions
Use of resources	HIGH	Patient makes explicit the value of the context elements as resources, using explicit criteria
	MEDIUM	Context elements are considered resources by the patient, without explicating criteria
	LOW	Patient does not refer to the context elements as possible resources
